# Glanzmann Thrombasthenia in a 14-Month-Old Infant: A Rare Platelet Function Disorder Presenting With Persistent Mucocutaneous Bleeding

**DOI:** 10.7759/cureus.108835

**Published:** 2026-05-14

**Authors:** Imane Chemlal, Hassnae Tkak, Manal Azizi, Ayad Ghanam

**Affiliations:** 1 Department of Pediatrics, Mohammed VI University Hospital, Faculty of Medicine and Pharmacy, Mohammed First University, Oujda, MAR

**Keywords:** bleeding gums, consanguinity marriage, glanzmann thromboasthenia, mucocutaneous bleeding, platelets function

## Abstract

Glanzmann thrombasthenia is a rare congenital platelet disorder affecting primary hemostasis, due to defective platelet aggregation and leading to mucocutaneous bleeding that can vary in severity. We report the case of a 14-month-old infant born to a first-degree consanguineous marriage, with a notable family history of unexplained severe bleeding in a maternal cousin. The patient was referred to our department for evaluation of mucocutaneous bleeding, with recurrent gingival hemorrhage and widespread ecchymoses. Initial hemostatic work-up was within normal ranges. Given the persistence of bleeding syndrome, platelet function analysis was performed, which demonstrated an aggregation defect consistent with Glanzmann thrombasthenia. This case emphasizes the diagnostic challenge of Glanzmann thrombasthenia in which routine coagulation studies are normal, and the need to maintain a high index of suspicion based on bleeding history, especially in consanguineous families. An early diagnosis is indispensable for the adequate treatment of the patient and to avoid life-threatening hemorrhagic complications.

## Introduction

Glanzmann thrombasthenia is a rare congenital bleeding disorder inherited in an autosomal recessive pattern, caused by a qualitative or quantitative deficiency of the platelet integrin αIIbβ3, which is essential for platelet aggregation, leading to early mucocutaneous bleeding in children [1]. Its incidence is higher in populations with a high rate of consanguinity, representing one per 200,000, which promotes the expression of homozygous forms [1,2].

Clinically, it is mainly characterized by epistaxis, gum bleeding, easy bruising, and prolonged bleeding following minor trauma [1,3]. These episodes may be recurrent and can lead to chronic anemia [1]. The diagnosis is based on normal routine hemostasis tests, including prothrombin time (PT) and activated partial thromboplastin time, which assess coagulation factors but not platelet function, associated with a prolonged bleeding time [1,4]. Platelet aggregation studies show absent responses to agonists (adenosine diphosphate (ADP), collagen, epinephrine) and preserved responses to ristocetin, reflecting a disorder of platelet function characterized by defective platelet aggregation and confirming the diagnosis [1,5]. Management relies on antifibrinolytic agents and platelet transfusions in cases of severe bleeding, with recombinant activated factor VIIa used in refractory cases [1,5].

We report the case of a 14-month-old infant born to consanguineous parents, with a family history suggestive of severe unexplained bleeding. The child presented with repeated gum bleeding and multiple ecchymoses. Initial hemostatic evaluation was normal. Further specialized platelet function testing ultimately confirmed the diagnosis of Glanzmann thrombasthenia. The interest of this case lies in its initial diagnostic challenge and in the limitations of standard coagulation tests in platelet function disorders and the importance of early clinical suspicion, especially in infants with a relevant family history and consanguinity. It also demonstrates that relying solely on the initial laboratory workup may delay diagnosis and underscores the importance of specific platelet function testing.

## Case presentation

A 14-month-old uncircumcised male infant, born to first-degree consanguineous parents with a family history of a maternal cousin’s death at age 19 from severe and unexplained bleeding, was referred to our pediatric hematology department for evaluation of a mucocutaneous bleeding syndrome. The presenting episode consisted of persistent moderate post-traumatic gingival bleeding lasting four hours, unresponsive to local compression and topical tranexamic acid administered before transfer. The episode was associated with multiple ecchymoses. The child also presented at an early age with recurrent, prolonged bleeding after vaccinations, spontaneous epistaxis, and mild gingival bleeding, all previously managed locally. The mother also reported frequent spontaneous bruising. There was no history of drug exposure, particularly non-steroidal anti-inflammatory drugs.

**Figure 1:**
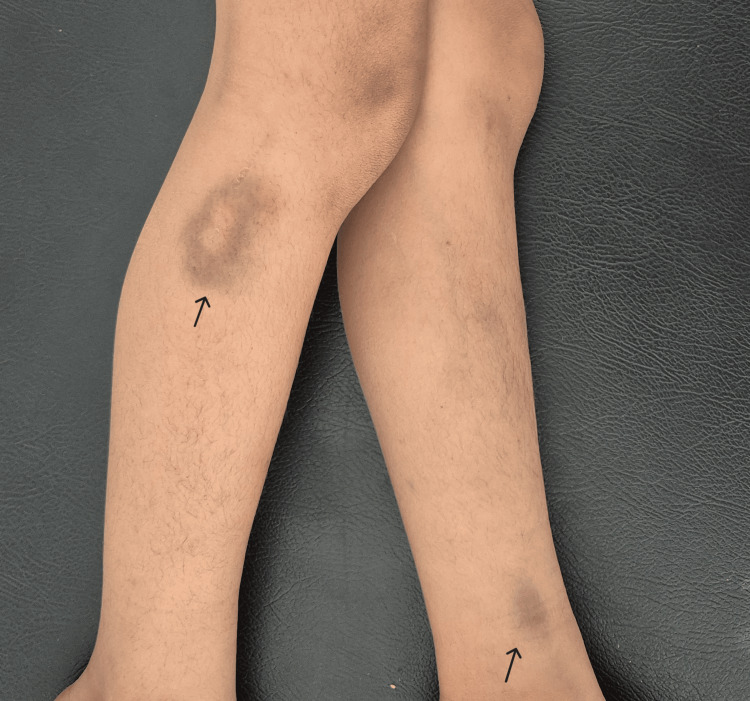
Ecchymoses (arrows) of the lower limbs in our case of Glanzmann thrombasthenia

On admission, the patient was alert and stable, with slightly pale conjunctivae. Physical examination revealed active gingival bleeding at the upper central incisor, a bruise on the lower lip, and multiple ecchymoses predominantly on the lower limbs (Figure 1), along with older lesions on the trunk. No hepatosplenomegaly or lymphadenopathy was detected, and the remainder of the examination was unremarkable. 

The family had undergone prior consultation for this cutaneous-mucosal bleeding syndrome at an early age. Prior investigations had shown microcytic hypochromic anemia, a normal platelet count, and normal PT and activated partial thromboplastin time (aPTT) ratio. Coagulation factor assays, including factors VIII and IX, were within normal limits. Based on these findings, iron supplementation was initiated.

At the current admission, laboratory tests (Table 1) confirmed persistent microcytic hypochromic anemia, a normal platelet count, and moderate leukocytosis. Standard coagulation parameters, including PT, aPTT, fibrinogen, and factors VIII and IX, were normal. von Willebrand factor levels were normal, excluding von Willebrand disease. The factor XIII assay was not performed due to financial constraints. Given persistent bleeding, a qualitative platelet function disorder was suspected. Platelet aggregation studies using light transmission aggregometry showed absent aggregation in response to all agonists except ristocetin, confirming the diagnosis of Glanzmann thrombasthenia.

**Table 1:** Laboratory findings of the patient ANC: absolute neutrophil count; aPTT: activated partial thromboplastin time; Hb: hemoglobin; Hct: hematocrit; MCH: mean corpuscular hemoglobin; MCHC: mean corpuscular hemoglobin concentration; MCV: mean corpuscular volume; PLT: platelets; PT: prothrombin time; vWF: von Willebrand factor; WBC: white blood cell count

Parameters	Result	Reference Range
Hb	9 g/dL	13-18 g/dL
Hct	29.20%	40-54%
MCV	74.70 fL	80-98 fL
MCH	23 pg	27-32 pg
MCHC	30.80%	32-36%
PLT	245,000 cells/µL	150,000-400,000 cells/µL
WBC	17,830 cells/µL	4,000-10,000 cells/µL
ANC	7,680 cells/µL	1,500-7,000 cells/µL
Lymphocytes	8,530 cells/µL	1,000-4,000 cells/µL
Monocytes	1,380 cells/µL	200-800 cells/µL
Eosinophils	180 cells/µL	0-500 cells/µL
Basophils	60 cells/µL	0-200/µL
PT	71%	70-100%
aPTT	1.12	< 1.20
Fibrinogen	2.50 g/L	2-4 g/L
Factor VIII	121.80%	70-150%
Factor IX	75%	70-120%
vWF activity	85.60%	50-150%
vWF antigen	83.20%	50-150%

Bleeding was controlled with local compression, combined with topical and systemic tranexamic acid. The patient was maintained on iron supplementation. The parents were counseled regarding bleeding risks, prevention, and emergency management, and close follow-up was scheduled. 

## Discussion

Glanzmann thrombasthenia is an autosomal recessive disorder of hemostasis caused by mutations in the ITGA2B or ITGB3 genes on chromosome 17q21 [1]. These disorders result in quantitative or qualitative abnormalities of the platelet αIIbβ3 (GPIIb/IIIa) receptor, leading to impaired platelet aggregation [1]. It is considered a very rare disorder worldwide, with an incidence of one per 1,000,000, but its incidence is higher in populations with a high rate of consanguinity, representing one per 200,000 [2]. Several studies have reported a slight female predominance [1,2], although 47% of cases are diagnosed before age two [3], and in some instances as early as the neonatal period [4].

In the present case, the infant was born to parents in a consanguineous marriage, a well-established risk factor for autosomal recessive disorders, including Glanzmann thrombasthenia, and the death of a maternal cousin from an unexplained bleeding disorder further suggests an inherited condition. In this context, especially in a consanguineous background, the key role of detailed family history in the diagnostic approach to pediatric bleeding disorders is emphasized, because suggestive familial bleeding patterns constitute an important argument in favor of Glanzmann thrombasthenia.

Glanzmann thrombasthenia usually presents with mucocutaneous bleeding, including especially easy bruising, epistaxis, gingival bleeding, and petechiae or ecchymoses, with a clinical spectrum ranging from mild to severe hemorrhage [1]. In pediatric patients, epistaxis is often the most frequent presenting symptom, while gingival bleeding is commonly associated with minor trauma or dental events [5,6]. Some hemorrhagic symptoms may go unnoticed until adulthood and only manifest during major hemostatic challenges, such as delivery or severe trauma. In adolescent females, menorrhagia is often underreported and constitutes a significant clinical concern because it can lead to iron deficiency anemia and markedly impair quality of life [7]. Although less common, gastrointestinal bleeding and other manifestations, such as intracranial hematomas, hematuria, and hemarthroses, may also occur and require careful clinical attention due to their potentially severe outcomes [7].

From a biological standpoint, Glanzmann thrombasthenia is characterized by a qualitative platelet function defect despite a normal platelet count, while standard coagulation tests, including PT and activated partial thromboplastin time, remain within normal ranges [1]. This dissociation between clinical bleeding and routine coagulation parameters may complicate diagnosis, particularly in young children, in whom hemorrhagic manifestations are often mild, intermittent, or nonspecific.

The reference test for diagnosing Glanzmann thrombasthenia remains light transmission platelet aggregation, which shows absent aggregation with all agonists except ristocetin. Flow cytometry is a complementary method that quantifies platelet GPIIb/IIIa (CD41/CD61) expression and allows classification into three subtypes [7]. Genetic testing of ITGA2B and ITGB3 confirms the diagnosis and guides genetic counseling [8]. In our case, further biological work-up was not performed because of resource limitations and refusal to undergo additional testing, including flow cytometry and genetic analysis. 

Treatment of Glanzmann thrombasthenia is based on both preventive strategies and targeted therapeutic interventions, tailored to bleeding severity and the clinical setting [9]. For minor hemorrhagic events, as well as for the prevention of bleeding in the setting of minor trauma or surgical procedures, local hemostatic measures associated with antifibrinolytic agents constitute the first-line treatment approach. For major bleeding or planned surgery, platelet concentrate administration remains the standard of care [9]. Nevertheless, therapeutic efficacy may be compromised by the development of alloantibodies in patients who have received multiple transfusions, resulting in platelet transfusion refractoriness that necessitates the use of recombinant activated factor VII [9]. In severe, refractory cases, hematopoietic stem cell transplantation (HSCT) is considered a curative approach. According to Wiegering et al., HSCT has been mainly performed in young children with a mean age of approximately five years, and has demonstrated favorable outcomes with all transplanted patients alive at follow-up [10]. Our case was diagnosed 14 months after post-traumatic gingival bleeding, underscoring the need for a high index of suspicion and a structured diagnostic approach. It also reinforces that platelet function disorders must be included in the differential diagnosis despite normal routine coagulation results.

## Conclusions

Glanzmann thrombasthenia should be considered early in children presenting with unexplained mucocutaneous bleeding, particularly in the context of consanguinity or suggestive family history, even when routine coagulation tests are normal. The present case reinforces the critical value of platelet function studies for diagnostic confirmation. Early detection facilitates timely management and improves both clinical outcomes and quality of life. A systematic genetic counseling for affected families is key to reducing the risk of recurrence in subsequent pregnancies.

## References

[1] Krause KA, Graham BC (2023). Glanzmann thrombasthenia. StatPearls.

[2] Khalifa GL, El-Sayed AA, Elmasry Z (2025). Epidemiological and clinical characteristics of children and young adults with Glanzmann's thrombasthenia in upper Egypt: a multicenter cross-sectional study. Ann Hematol.

[3] Khair K, Fletcher S, Boyton M, Holland M (2024). Bleeding and quality of life in people with Glanzmann thrombasthenia-insights from the Glanzmann's 360 study. Res Pract Thromb Haemost.

[4] Alyami NH, Musallam SH, Al Greshah H (2026). Neonatal Glanzmann’s thrombasthenia presenting as refractory post-circumcision hemorrhage in a region of high consanguinity: a case report. Cureus.

[5] Sherief LM, El Ekiaby M, El-Hawy M, Elhawary E, Nazim AA, Elbahy SM (2025). Glanzmann thrombasthenia: a multi-center study of demographics, clinical spectrum, and treatment efficacy. Eur J Pediatr.

[6] Toygar HU, Guzeldemir E (2007). Excessive gingival bleeding in two patients with Glanzmann thrombasthenia. J Periodontol.

[7] Mathews N, Rivard GE, Bonnefoy A (2021). Glanzmann thrombasthenia: perspectives from clinical practice on accurate diagnosis and optimal treatment strategies. J Blood Med.

[8] Solh T, Botsford A, Solh M (2015). Glanzmann's thrombasthenia: pathogenesis, diagnosis, and current and emerging treatment options. J Blood Med.

[9] Fiore M, Giraudet JS, Alessi MC (2023). Emergency management of patients with Glanzmann thrombasthenia: consensus recommendations from the French reference center for inherited platelet disorders. Orphanet J Rare Dis.

[10] Wiegering V, Sauer K, Winkler B, Eyrich M, Schlegel PG (2013). Indication for allogeneic stem cell transplantation in Glanzmann's thrombasthenia. Hamostaseologie.

